# NF-κB in inflammation and renal diseases

**DOI:** 10.1186/s13578-015-0056-4

**Published:** 2015-11-16

**Authors:** Haisong Zhang, Shao-Cong Sun

**Affiliations:** Department of Nephrology, Affiliated Hospital of Hebei University, No. 213 Yuhuadonglu, Baoding, 071000 China; Department of Immunology, The University of Texas MD Anderson Cancer Center, 7455 Fannin Street, Box 902, Houston, TX 77030 USA; The University of Texas Graduate School of Biomedical Sciences at Houston, Houston, TX 77030 USA

**Keywords:** NF-κB, Inflammation, Renal diseases, Nephritis, IgA nephropathy

## Abstract

Nuclear factor κB (NF-κB) is a family of inducible transcription factors that plays a vital role in different aspects of immune responses. NF-κB is normally sequestered in the cytoplasm as inactive complexes via physical association with inhibitory proteins termed IκBs. In response to immune and stress stimuli, NF-κB members become activated via two major signaling pathways, the canonical and noncanonical pathways, and move to the nucleus to exert transcriptional functions. NF-κB is vital for normal immune responses against infections, but deregulated NF-κB activation is a major cause of inflammatory diseases. Accumulated studies suggest the involvement of NF-κB in the pathogenesis of renal inflammation caused by infection, injury, or autoimmune factors. In this review, we discuss the current understanding regarding the activation and function of NF-κB in different types of kidney diseases.

## Background

**Fig. 1:**
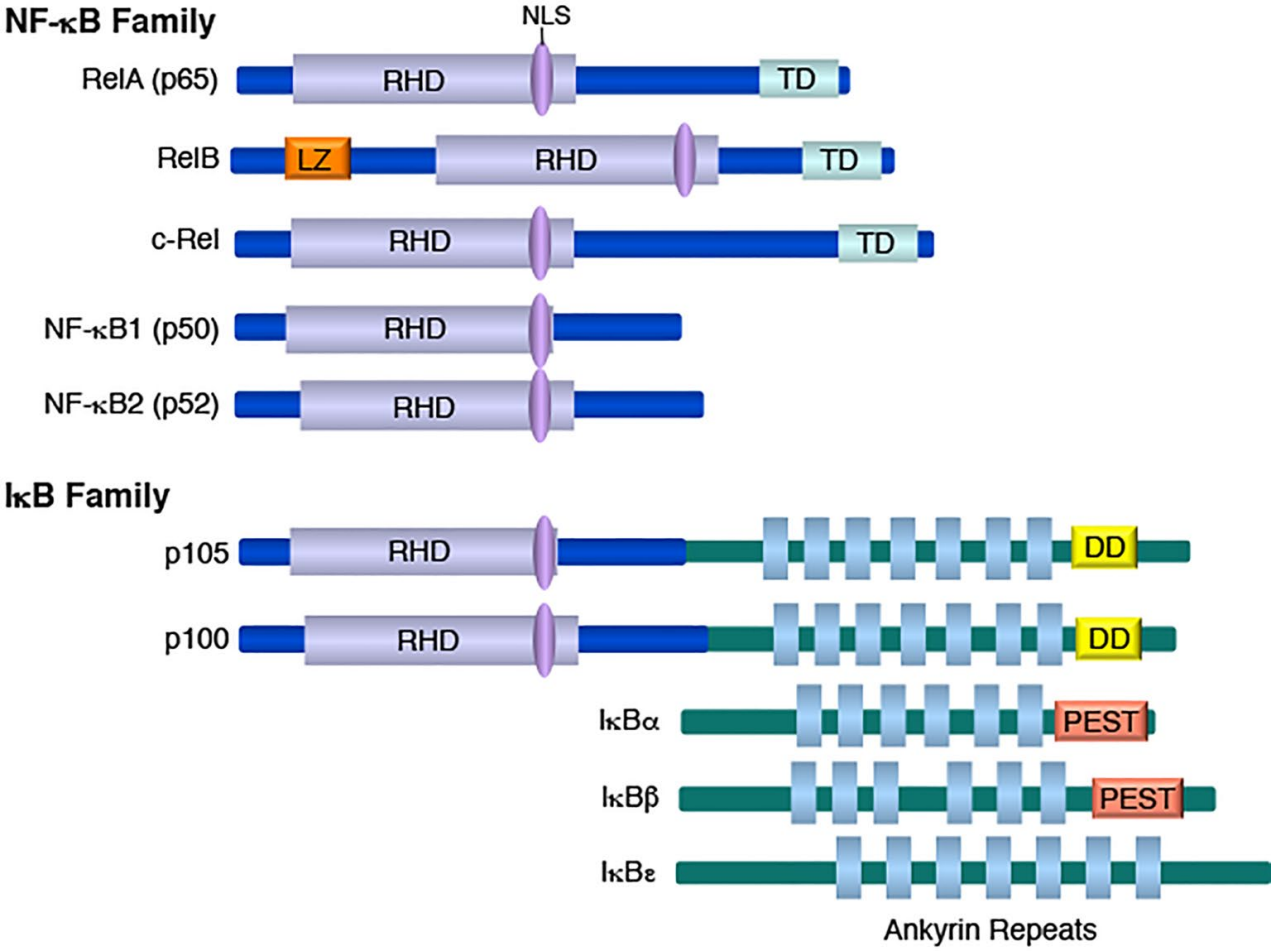
The mammalian NF-κB and IκB families. The five members of the NF-κB family are schematically shown, with the major domains highlighted and the alternative names indicated in parenthes. The rel-homology domain (RHD) mediates DNA-binding and dimerization functions, the transactivation domain (TD) is required for transcriptional activation of target genes, whereas the leucine zipper (LZ) motif is also involved in target gene transactivation. The IκB family includes the p50 precursor protein p105, the p52 precursor protein p100, IκBα, IκBβ, IκBε, and several atyipical IκB members that are not shown in the figure. A hallmark of IκB members if the presence of ankyrin repeats that are required for inhibition of NF-κB. The death domain (DD) of p105 and p100 is also important for their IκB-like functions. The PEST (proline, glutamine, serine, and threonine)-like sequence of IκBα and IκBβ mediates protein turnover. *RHD* rel-homology domain, *TD* transactivation domain, *LZ* leucine zipper, *DD* death domain, *PEST* proline, glutamine, serine, and threonine

Nuclear factor κB (NF-κB) was initially discovered as a B cell nuclear protein binding to the κ enhancer of the immunoglobulin κ light chain gene [1, 2]. It subsequently became clear that NF-κB is a ubiquitously expressed transcription factor that mediates signal-induced expression of numerous genes involved in different biological processes, including immune responses, inflammation, cell growth and survival [3, 4]. Mammalian NF-κB represents a family of structurally related proteins, including RelA (also called p65), RelB, c-Rel, p50 (also called NF-κB1), and p52 (also called NF-κB2), which share extensive homology in a region known as Rel homology domain (Fig. 1). Through this domain, the different NF-κB members interact to form various homo- and hetero-dimers and bind to κB sequence elements present in the promoter or enhancer regions of target genes [4]. Each of the Rel proteins (RelA, RelB, c-Rel) contains a C-terminal transactivation domain, required for inducing target gene transcription. p50 and p52 lack a transactivation domain and functions to modulate the DNA-binding activity of NF-κB by forming Rel/p50 and Rel/p52 heterodimers. The homodimers of p50 and p52 are transcriptional repressors that play an important role to prevent aberrant expression of NF-κB target genes, including those involved in inflammation [5–8]. However, the p50 and p52 homodimers may also acquire transactivation function by associating with non-Rel coactivator proteins [9, 10].

NF-κB dimers are normally sequestered in the cytoplasm as inactive complexes via physical interaction with inhibitory proteins termed IκBs (Fig. 1). A hallmark of IκBs is the presence of an ankyrin-repeat domain, which is required for interacting with NF-κB and inhibiting the nuclear translocation and DNA binding activity of NF-κB dimers. The most extensively studied member of the IκB family is IκBα, which is vital for controlling the function of the prototypical NF-κB dimer, RelA/p50 [11]. Several other IκB molecules have been characterized, including IκBβ, IκBε, and several atypical IκB proteins [3, 4]. The IκB family also includes p105 and p100, precursor proteins of NF-κB1 and NF-κB2, respectively [12]. These precursor proteins contain, in their C-terminal portion, an IκB-like structure and, thus, function as inhibitors of NF-κB, belonging to the IκB family (Fig. 1). Generation of mature NF-κB1 (p50) and NF-κB2 (p52) involves proteasome-mediated degradation of the IκB-like sequence of p105 and p100. Thus, this so-called processing of p105 and p100 not only generate mature NF-κB1 and NF-κB2 but also disrupts the IκB-like function of these NF-κB precursor proteins [12–14].

The in vivo functions of NF-κB members have been extensively studied by gene-targeting approaches in mice. Despite their structural homology and DNA-binding similarities, the different NF-κB members have both overlapping and different functions in vivo [15]. Similarly, gene-targeting studies have revealed different functions of the IκB family members. These findings highlight the complexity of this transcription factor system.

### NF-κB signaling pathways

**Fig. 2:**
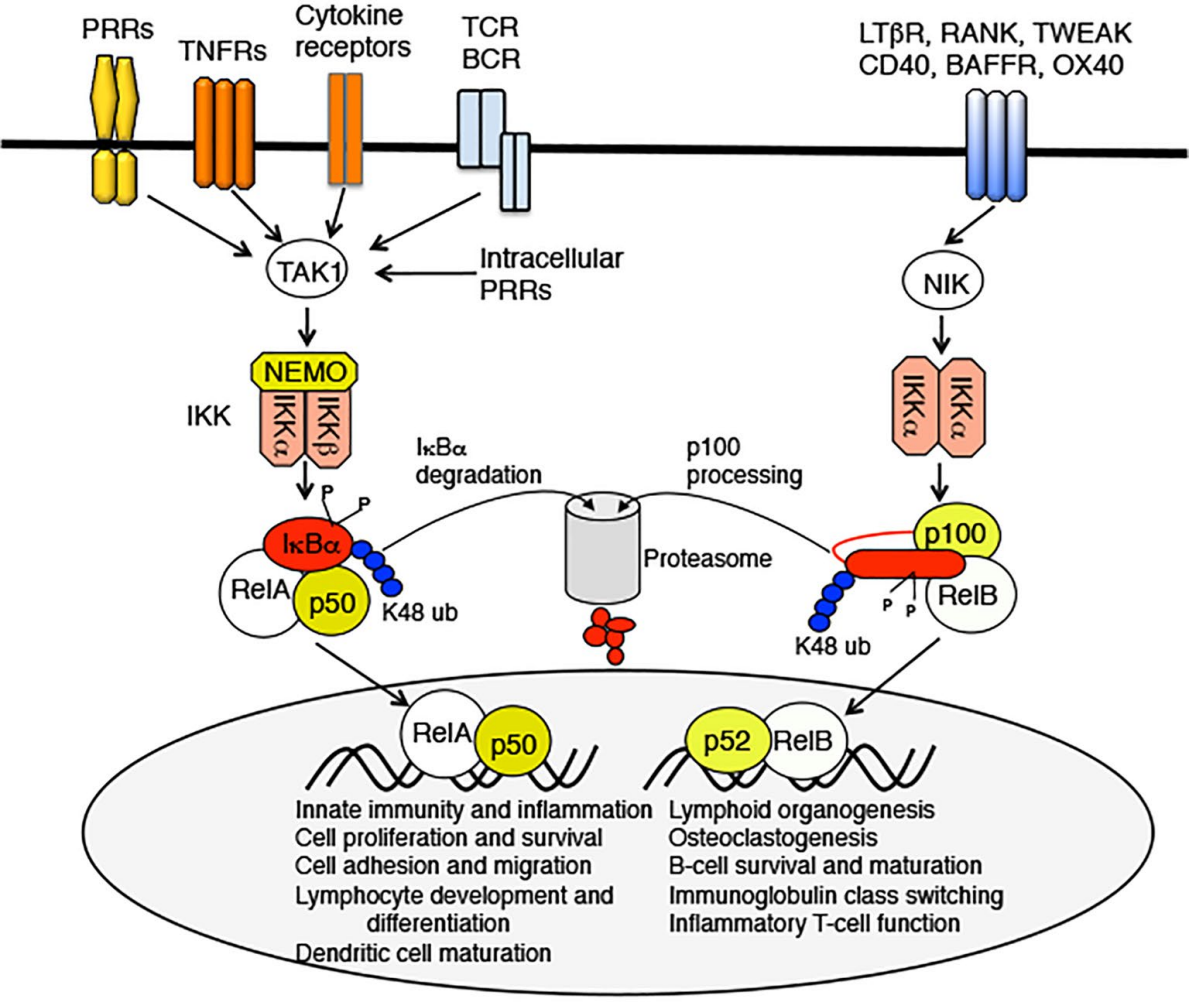
Canonical and noncanonical NF-κB signaling pathways. The canonical NF-κB pathway responds to signals from diverse receptors, including pattern-recognition receptors (PRRs) present on cell surface or intracellular environment, TNF receptors (TNFRs), other cytokine receptors, as well as T cell receptor (TCR) and B cell receptor (BCR). The noncanonical NF-κB pathway is activated by a selective subset of TNFR superfamily members. Canonical NF-κB signaling involves activation of the trimeric IKK complex by the MAP3 K TAK1, IKK-mediated IκBα phosphorylation and subsequent degradation, and nuclear translocation of the prototypical NF-κB heterodimer RelA/p50. Noncanonical NF-κB signaling relies on NF-κB inducing kinase (NIK), which together with IKKα mediate phosphorylation and processing of p100, causing generation of p52 and nuclear translocation of p52/RelB complex. Compared with the pleotropic roles of canonical pathway, noncanonical NF-κB has more specific functions. *PRR* pattern-recognition receptors, *TNFR* TNF receptor, *TCR* T cell receptor, *BCR* B cell receptor, *NIK* NF-κB inducing kinase

There are two major signaling pathways that mediate NF-κB activation: the canonical and noncanonical pathways [3, 13] (Fig. 2). The canonical pathway relies on a multi-subunit IκB kinase (IKK), composed of two catalytic subunits, IKKα and IKKβ, and a regulatory subunit named NF-κB essential modulator (NEMO) or IKKγ [3, 4]. IKK responds to various cellular stimuli, including microbial components, cytokines, growth factors and mitogens, and agents causing stress. Upon activation, IKK phosphorylates IκB and, thereby, triggers ubiquitin-dependent IκBα degradation and release of the sequestered NF-κB members, including RelA/p50 and c-Rel/p50 dimers. The major IκB member regulating canonical NF-κB pathway is IκBα, a protein characterized by its dynamic changes along with signal-induced NF-κB activation. Following its degradation triggered by IKK-mediated phosphorylation, IκBα is rapidly resynthesized via NF-κB-mediated induction of its gene expression, thus providing a feedback mechanism to terminate NF-κB responses in a timely manner [16, 17].

Activation of IKK and canonical NF-κB signaling by most cellular stimuli requires TGFβ-activated kinase 1 (TAK1), a member of the MAP kinase kinase kinase (MAP3K) family that directly phosphorylates the activation loop of IKKβ [18]. A hallmark of TAK1 and IKK activation is the involvement of lysine 63 (K63)-linked ubiquitination [19]. It is generally believed that cellular stimuli induce the conjugation of ubiquitin chains to signaling adaptors, which facilitate the recruitment of TAK1 and IKK. Both TAK1 and IKK contain a ubiquitin-binding subunit, TAB 2 and NEMO, respectively, and the ubiquitin association not only facilitates assembly of the TAK1/IKK signaling complex but may also directly contribute to the catalytic activation of these kinases [19]. Accumulating studies suggest that TAK1 and NEMO are also conjugated with ubiquitin chains, which contributes to their activation [11, 19, 20]. In addition to K63-linked ubiquitin chains, linear ubiquitin chains (also called M1-linked ubiquitin chains) are also involved in the activation of IKK by certain inducers [21, 22]. Linear ubiquitin chains are catalyzed by a ubiquitin assembly complex, LUBAC, composed of heme-oxidized IRP2 ubiquitin ligase-1 (HOIL-1, also called RBCK1), HOIL-1-interacting protein (HOIP, also called RNF31), and the adaptor protein SHANK-associated RH domain-interacting protein (SHARPIN). LUBAC conjugates linear ubiquitin chains onto NEMO in TNF receptor (TNFR) signaling pathway, which promotes IKK activation and stabilization of the TNFR signaling complex [23, 24]. NEMO also binds to linear ubiquitin chains, which is important for TNF-induced NF-κB activation [25]. Another signaling factor that is conjugated with linear ubiquitination in the TNFR pathway is the adaptor RIP1, which is important for NF-κB activation and inhibition of TNF-induced cell death [26].

Tight control of ubiquitination is crucial for maintaining the homeostatic and signal-induced activation of NF-κB [27]. In particular, the canonical NF-κB signaling pathway is negatively regulated by ubiquitin-specific proteases, or deubiquitinases, such as CYLD and A20 [27]. CYLD deconjugates ubiquitin chains from a number of signaling molecules, including NEMO, TAK1, RIP1, TRAF2, and TRAF6 [28]. Gene-targeting studies have revealed a crucial role for CYLD in controlling the homeostatic NF-κB activation in lymphocytes, a function that is in turn required for maintaining normal functions of T and B cells and preventing autoimmunity and inflammation [20, 28, 29]. CYLD specifically regulates non-degradative types of ubiquitination by preferentially cleaving K63-linked and linear ubiquitin chains [30]. A20 functions as a pivotal feedback regulator of signal-induced canonical NF-κB activation [27, 31]. Upon activation, NF-κB induces the expression of A20, and the accumulated A20 inhibits activation of IKK and NF-κB. A unique feature of A20 is that it possesses both K63-specific DUB activity and K48-specific E3 ligase function [32]. This property allows A20 to mediate ubiquitin editing by cleaving K63-linked ubiquitin chains and conjugating K48-linked ubiquitin chains to substrates, such as RIP1 and Ubc13, thereby both inhibiting the signaling function and triggering proteosomal degradation of the substrates [32]. However, the function of A20 appears to be complex, since a recent study reveal that knockin mice expressing a DUB-inactive A20 mutant have no defect in NF-κB activation by TNF or LPS, suggesting a DUB-independent function of A20 [33].

The noncanonical NF-κB pathway does not require the trimeric IKK complex or IκBα degradation but rather depends on the processing of the *Nfkb2* gene product p100 [13, 14] (Fig. 2). P100 is the precursor protein of the NF-κB subunit p52, which contains a C-terminal portion that is homologous to IκBs (Fig. 1). Like IκBs, p100 binds to NF-κB members and functions as an NF-κB inhibitor [34]. The processing of p100 involves selective degradation of its C-terminal IκB-like sequence, which not only generates p52 but also leads to nuclear translocation of its sequestered NF-κB members, predominantly RelB [14]. A central signaling component of the noncanonical NF-κB pathway is NF-κB inducing kinase (NIK), which functions together with a downstream kinase, IKKα, to induce phosphorylation-dependent p100 processing [35, 36]. To date, the well-defined receptors that induce noncanonical NF-κB signaling are a subset of TNFR superfamily members, such as lymphotoxin beta receptor (LTbR), B cell-activating factor belonging to the TNF family (BAFF, also called BLyS) receptor (BAFFR), CD40, receptor activator of nuclear factor-kappaB (RANK), and tumor necrosis factor-related weak inducer of apoptosis (TWEAK) [14].

In contrast to the rapid and transient nature of canonical NF-κB signaling, the noncanonical NF-κB signaling pathway is characteristically slow and persistent [13]. This is largely due to the unusual mechanism underlying NIK activation. The steady level of NIK is normally low due to its constant degradation by a TRAF3-dependent uibuiqination mechanism, and induction of noncanonical NF-κB signaling involves stabilization and accumulation of NIK as a result of TRAF3 degradation [37]. This mechanism of NIK regulation also involves TRAF2 and the E3 ubiquitin ligase c-IAP (c-IAP1 or c-IAP2). These components appear to form an E3 complex, in which TRAF3 functions as a substrate-binding subunit and TRAF2 functions as an adaptor recruiting c-IAP to TRAF3 and NIK [14]. The TRAF3-TRAF2-cIAP complex controls the steady state function of NIK and noncanonical NF-κB signaling. Signal-induced noncanonical NF-κB activation is also subject to regulation by negative regulators. A deubiquitinase, Otud7b (also called Cezanne) inhibit signal-induced ubiquitination and degradation of TRAF3, thereby negatively regulating the induction of p100 processing by TNFR family members in B cells and fibroblasts [38]. In addition, NIK is negatively regulated by two homologous kinases, IKKα and TBK1 [39, 40]. These kinases phosphorylate NIK and promote degradation of NIK even when it is released from TRAF3.

### NF-κB in inflammation

Inflammation is a body’s protective response to infections and tissue damages, characterized by vasodilation and recruitment of leukocytes, plasma proteins and fluid to the affected tissue [41, 42]. Inflammation is normally beneficial to the host; however, deregulated inflammatory responses can cause excessive or long-lasting tissue damages, leading to acute or chronic inflammatory diseases. The development of an inflammation is typically initiated through the detection of pathogen-associated molecular patterns (PAMPs) or damage-associated molecular patterns (DAMPs) by pattern-recognition receptors (PRRs) on innate immune cells and epithelial cells. PRRs represent several families of receptors, including toll-like receptors (TLRs), RIG-I like receptors (RLR), NOD-like receptors (NLRs), and C-type lectin like receptors (CLRs), which are expressed either on the surface or intracellular environments of the host cells [43]. Upon ligation, PRRs initiate intracellular signaling events that lead to induction of proinflammatory cytokines, chemokines, and other inflammatory mediators.

Common to the signaling events elicited by the different PRRs is activation of the canonical NF-κB pathway, which mediates transcriptional induction of various proinflammatory cytokines, such as TNF-α, IL-1, and IL-6, as well as a number of chemokines [43]. These soluble factors bind to their specific receptors to induce important inflammatory processes, including vasodilation and recruitment of monocytes and neutrophils to the site of inflammation [42]. NF-κB is also a pivotal mediator of signal transduction stimulated by several major inflammatory cytokines, such as TNF-α and IL-1, thereby participating in the effector phase of inflammation [44]. Indeed, studies based on animal models and human patients suggest the involvement of NF-κB in the pathogenesis of various inflammatory diseases [44].

Inflammation also involves the adaptive immune components, including specific subsets of T helper (Th) cells derived from activated CD4^+^ T cells [42]. Upon activation, naïve CD4^+^ T cells differentiate into different subsets of effector T cells, including Th1, Th2, Th17, and T follicular (Tfh) cells, which secrete distinct cytokines and mediate different aspects of immune responses [45]. In addition, activated CD4^+^ T cells also produce a subset of immunosuppressive T cells, the inducible T regulatory (Treg) cells. The cytokines secreted by innate immune cells play a crucial role in regulating the differentiation of CD4^+^ T cells, providing a link between innate and adaptive immune responses. Th1 and Th17 cells are considered proinflammatory T cells because of their association with autoimmune and inflammatory conditions [42]. Th1 cells are characterized by secretion of interferon gamma (IFNγ), a cytokine with pivotal functions in cellular immunity and inflammatory responses. The signature cytokine of Th17 cells is IL-17, which is linked with many autoimmune and inflammatory diseases [46, 47]. Notably, NF-κB is required for production of both Th1 and Th17 cells [48]. NF-κB functions in innate immune cells to induce the production of IL-12 and IL-23, which in turn promote the differentiation of CD4^+^ T cells to Th1 and Th17 cells, respectively [49]. Canonical NF-κB also has a T cell-intrinsic role in mediating the generation of Th1 and Th17 cells [50–52]. The NF-κB members RelA and c-Rel are required for TCR-stimulated expression of *Rorc*, a gene encoding the Th17-lineage specific transcription factors RORγT and RORγ [51, 52]. NF-κB not only promotes induction of Th17 cells but also serves as a major transcription factor that is actived by IL-17 and mediates the inflammatory functions of Th17 cells [53, 54].

Recent studies suggest that the noncanonical NF-κB pathway also plays a role in regulating inflammatory responses. Although noncanonical NF-κB pathway is dispensable for CD4^+^ T cell differentiation, this pathway is required for the inflammatory effector function of Th17 cells [55]. After migrating to the inflammatory microenvironment, Th17 cells acquire pathological effector functions by expressing specific cytokines including GM-CSF [56, 57]. Genetic evidence suggests a crucial role for the noncanonical NF-κB pathway in mediating induction of GM-CSF in Th17 cells [55]. The noncanonical NF-κB member p52 directly binds to the κB enhancer element in the GM-CSF gene promoter and recruits the canonical NF-κB member c-Rel to this promoter. Thus, p52 and c-Rel synergize in the transcriptional activation of the GM-CSF gene, which promotes inflammation in an animal model of neuroinflammatory disease [55]. In summary, both the canonical and noncanonical NF-κB pathways are linked to inflammation, although they act with different mechanisms.

### Inflammation and kidney diseases

Immune and inflammatory factors play an important role in the pathogenesis of kidney diseases [58, 59]. Innate immune cells, such as macrophages and dendritic cells, are thought to have an important role in mediating renal inflammation and injury [59]. During an in infection, these innate immune cells detect microbial products via PRRs, such as TLRs, and are stimulated to secrete proinflammatory cytokines and chemokines [59, 60]. In addition to recognizing PAMPs, the TLRs also respond to DAMPs, which are endogenous ligands generated by tissue damage. DAMPs serve as an important trigger for innate immune cell activation and inflammation in the kidney. In addition to innate immune cells, renal cells, such as mesangial and tubular epithelial cells, also express TLRs and produce proinflammatory cytokines and chemokines, contributing to kidney injury [60, 61].

In addition to infections and injuries, autoimmune disorders represent a major cause of renal inflammation and injury. Lupus nephritis is caused by the autoimmunity systemic lupus erythematosus (SLE; often called lupus) [62, 63]. The pathogenesis of SLE and lupus nephritis involves both adaptive and innate immune cells, including T cells, B cells, dendritic cells and macrophages, as well as renal cells [59, 62, 64, 65]. Lupus nephritis is characterized by the presence of various autoantibodies that form immune complexes and deposit to kidney glomeruli [63]. The immune complexes induce inflammatory responses through a number of mechanisms, such as activation of complement and Fc receptors and recruitment of inflammatory cells [66, 67]. Immune complexes also serve as an endogenous trigger of TLRs on renal cells to induce expression of proinflammatory cytokines [60]. IgA nephropathy, a leading cause of primary glomerulonephritis, is also considered an autoimmune disease [68]. Patients with IgA nephropathy produce high levels of aberrantly glycosylated IgA and anti-glycan autoantibodies, leading to the formation of IgA-immune complexes deposited to the kidney glomeruli and progressive induction of kidney injury [68].

### NF-κB activation by pathophysiological triggers in renal cells

NF-κB is activated by different pathophysiological triggers in renal cells and has been linked to experimental and human kidney diseases [69–71]. Microbial components, such as LPS of Gram-negative bacteria, are strong stimuli of NF-κB in kidney resident cells and infiltrating immune cells [69]. Outer membrane proteins of leptospira, pathogens associated with renal diseases, also activate NF-κB and act through stimulation of TLR2 [72]. Cytokines and other pathological factors produced during renal ischaemia–reperfusion injury are also strong stimulators of NF-κB. In particular, ischaemia–reperfusion induces the production of TNF-α in an NF-κB-dependent manner, and TNF-α in turn binds to its receptor to stimulate NF-κB activation, thereby providing a positive feedback mechanism of NF-κB regulation [73]. This signaling loop plays an important role in the pathogenesis of renal ischemia–reperfusion injury. Angiotensin II, a peptide hormone overproduced during renal damage, has been shown to activate NF-κB [74, 75]. Angiotensin is a physiological regulator of vasoconstriction and blood pressure; however, deregulated angiotensin is involved in inflammation and the pathogenesis of hypertension, atherosclerosis, and cardiac and renal injuries [76]. NF-κB activation plays an important role in angiotensin II-induced expression of chemokines and inflammatory responses in renal injury [77–79].

Some other pathological agents associated with kidney diseases are also inducers of NF-κB. For example, it has been shown that aberrantly glycosylated IgA, pathological agents of IgA nephropathy, activates NF-κB in mesangial cells by modulating proteasome function [80, 81]. Another potential pathological trigger of NF-κB activation is hyperhomocysteinemia [82], a condition characterized by abnormal elevation of plasma homocysteine levels and seen in chronic disorders including experimental kidney diseases [83, 84]. In a rat model, diet-induced hyperhomocysteinemia was shown to induce IκBα phosphorylation and canonical NF-κB activation in kidney, which is responsible for the induction of the inflammatory mediator iNOS [82]. In vitro studies also reveal that homocysteine activates NF-κB, which contributes to chemokine induction in macrophages and smooth muscle cells [85, 86].

As seen in other tissues, NF-κB activation in kidney cells is negatively regulated by different factors. Nephrin, an Ig superfamily member located at the slit diaphragm of glomerular podocytes, serves as a negative regulator of NF-κB signaling in podocytes [87]. Although precisely how nephrin inhibits NF-κB activation is incompletely understood, it appears to involve inhibition of the atypical PKC aPKCζ. Uncontrolled NF-κB activation in kidney podocytes appears to contribute to the glomerular injury [87]. Another negative regulator of NF-κB in kidney cells is the deubiquitinase Cezanne (also called Otud7b), which regulates the inflammatory responses in glomerular endothelial cells by controlling the ubiquitination and function of TRAF6 [88]. Cezanne is a DUB that shares homology with A20 in the catalytic domain [89]. Cezanne negatively regulates canonical or noncanonical NF-κB pathways, depending on the cell types. Cezanne expression is induced in multiple cell types of murine kidneys exposed to ischemia–reperfusion. Genetic ablation of Cezanne in mice enhances renal inflammation and injury induced by ischemia–reperfusion [88]. Loss of Cezanne increased the induction of VCAM-1 and E-Selection as well as RelA phosphorylation [88]. Since the expression of these cell adhesion molecules is also regulated by noncanonical NF-κB, which is negatively controlled by Cezanne, it will be interesting to examine whether noncanonical NF-κB is also involved in the regulation of renal inflammation mediated by Cezanne. The proinflammatory function of NF-κB in renal cells is also subject to regulation by the p50/p50 homodimer, a κB-specific repressor that is induced during experimental renal injury and serves as a feedback repressor of NF-κB-mediated inflammatory gene induction [90, 91].

### NF-κB in kidney injury

Acute kidney injury (AKI) is a frequently seen kidney disease associated with a high rate of morbidity and mortality, and survivors of AKI faces a long-term risk for developing chronic kidney disease [92, 93]. AKI is often caused by ischemia–reperfusion, during which kidney is in a condition of hypoxia and low renal blood flow. Inflammation caused by AKI is an important factor that exacerbates kidney injury, and control of inflammation has proved to be effective for minimizing kidney injury and facilitating recovery [94]. NF-κB is activated along with kidney injury induction by ischemia–reperfusion and believed to serve as an important mediator of inflammation [71]. It has been shown that NF-κB inhibitors attenuate the induction of renal inflammation and injury in animal models [95, 96]. Inhibitor studies also suggest a role for NF-κB in regulating aldosterone/salt-induced renal injury [97]. A more recent study tested the effect of a small interfering RNA (siRNA) for IKKβ on renal injury. In a rat kidney injury model, administration of IKKβ siRNA via renal artery injection inhibits IKKβ expression and NF-κB activation, which is associated with diminished kidney injury and inflammation induced by ischemia–reperfusion [98].

A recent study reveals that induction of acute kidney injury by high doses of folic acid is associated with increased expression of NF-κB members, RelA and NF-κB2 [99]. Inhibition of NF-κB with an inhibitor, pyrrolidine dithio-carbamate ammonium (PDTC) ameliorated the kidney dysfunction, suggesting that NF-κB plays a role in the pathogenesis of kidney injury. NF-κB has also been implicated in the pathogenesis of kidney damage caused by hypertension, a chronic medical condition with repeatedly elevated blood pressure [100, 101]. The elevated level of angiotensin II, associated with hypertension, is a trigger for NF-κB activation and induction of inflammatory responses. Inhibition of NF-κB by transgenic expression of a degradation-resistant IκBα mutant, IκBα∆N, in endothelial cells ameliorates renal injury caused by hypertension in a mouse model. The NF-κB inhibition does not influence induction of hypertension but rather blocks hypertension-mediated induction of proinflamatory cytokines and cell adhesion molecules involved in renal damages [100]. Similar results were obtained using a rat model of hypertension, in which an NF-κB inhibitor, PDTC, inhibits angiotensin II-induced inflammatory renal damage [101].

### NF-κB in IgA nephropathy

IgA nephropathy is the most commonly seen form of glomerulonephritis and is caused by aberrant production of glycosylated IgA and its deposition to the kidney glomeruli as immune complexes [102, 103]. It has long been known that binding of IgA to Fc alpha receptors on mesangial cells activates NF-κB, which contributes to the induction of the chemokines MCP-1 and IL-8 [80]. Moreover, elevated levels of NF-κB have been detected in the tubular area of patients with IgA nephropathy, which is correlated with poor disease outcome [104–106]. NF-κB inhibitors have been implicated as anti-inflammatory agents for the treatment of immune glomerulonephritis [107, 108].

The noncanonical NF-κB has also been implicated in the regulation of IgA nephropathy [39]. Genetic deficiency in a negative regulator of the noncanonical NF-κB pathway, TBK1, causes IgA hyper-production and development of nephropathy-like symptoms in mice [39]. The TBK1-knockout mice have a substantially increased level of serum IgA and antibody deposition in the kidney glomeruli, associated with symptoms of kidney dysfunctions, such as increased levels of urinary protein and serum nitrogen and creatinine [39]. The noncanonical NF-κB pathway is crucial for the induction of IgA class switching by CD40 and BAFFR, and TBK1 controls IgA class switching via negative regulation of noncanonical NF-κB activation [39]. Consistently, transgenic mice overexpressing the noncanonical NF-κB inducer BAFF in B cells aberrantly produce IgA and develop IgA nephropathy [109, 110].

### NF-κB in lupus nephritis

Lupus nephritis is a frequently seen complication in patients with SLE and is known to significantly reduce the survival of SLE patients [111]. A hallmark of lupus nephritis is the renal inflammation caused by deposition of autoimmune complexes to kidney glomeruli [65]. NF-κB has been implicated in the pathogenesis of lupus nephritis. Patients with lupus nephritis have elevated expression and activation of NF-κB in glomerular endothelial and mesangial cells, coupled with upregulation of inflammatory cytokines [112, 113]. Inhibition of IKKβ attenuates the induction of inflammatory mediators by hypoxia in rat renal tubular cells [114]. An IKK-selective inhibitor, Bay11-7082, ameliorates a mouse model of lupus nephritis by inhibiting NF-κB and the inflammasome NLRP3 [115]. Consistently, the genes encoding two NF-κB-negative regulators, A20 (also called TNFAIP3) and A20-binding inhibitor of NF-κB1 (ABIN1; also called TNIP1), have been associated with human lupus and lupus nephritis [116, 117]. A20 is a ubiquitin-editing enzyme that negatively regulates NF-κB activation by various immune and inflammatory stimuli [118, 119], and ABIN1 is a ubiquitin-binding protein that inhibits NF-κB signaling by probably facilitating the action of A20 and, thereby, interfering with signal-induced activation of IKK [120]. A20 deficiency in both human patients and animal models are associated with autoimmune and inflammatory diseases, including lupus [121]. Both human and mouse genetic studies also suggest the involvement of ABIN1 in autoimmune nephritis. In particular, knockin mice expressing an inactive form of ABIN1 display aberrant activation of NF-κB and develop lupus-like autoimmunity and pathological symptoms resembling human lupus nephritis [117, 122].

A recent study suggests that Nrf2 regulates lupus nephritis via inhibition of both oxidative injury and NF-κB activation [123]. Nrf2 is a basic leucine zipper transcription factor with a crucial cytoprotective role in cellular responses to oxidative stress [124]. Nrf2 mediates transcription of genes encoding antioxydants and other cytoprotective factors. Recent work suggests that the anti-inflammatory function of Nrf2 may also involve inhibition of NF-κB [123, 125], although the underlying mechanism is elusive. Mice deficient in Nrf2 spontaneously develop lupus-like autoimmune nephritis at old ages [126]. In a pristane-induced experimental lupus nephritis model, the Nrf2-deficient mice develop more severe renal damage and pathological symptoms [123]. Activation of NF-κB appears to be responsible for the aberrant production of inflammatory mediators, such as ROS and iNOS, and disease symptoms in the knockout mice.

### NF-κB in TWEAK-stimulated inflammation in kidney diseases

TWEAK is a member of the TNF superfamily of cytokines [127]. TWEAK induces signal transduction via binding to its receptor, fibroblast growth factor-inducible 14 (Fn14). Strong evidence suggests that TWEAK plays an important role in the pathophysiological processes of kidney diseases [128, 129]. TWEAK is expressed by infiltrating myeloid cells and T cells as well as renal tubular epithelial cells and mesangial cells. The TWEAK receptor Fn14 is expressed in several cell types of the kidney, including tubular cells, mesangial cells, and podocytes, as well as infiltrating macrophages [130]. Human kidney disease patients and animal models of kidney injury are associated with increased expression of TWEAK and Fn14 [129, 131, 132]. TWEAK stimulates the activation of both canonical and noncanonical NF-κB pathways in renal tubular cells, thereby inducing the production of proinflammatory mediators [133, 134]. TWEAK induces the expression of proinflammatory cytokines and chemokines (such as MCP-1 and Rantes) via the canonical NF-κB pathway and the chemokines CCl21 and CCL19 via the NIK-dependent noncanonical NF-κB pathway [128]. Consistently, genetic ablation of TWEAK renders mice resistant to the induction of renal inflammation and fibrosis, whereas overexpression of TWEAK promotes the induction of kidney obstructions [135]. Genetic deficiency in Fn14 also ameliorates lupus nephritis in both induced and spontaneous models [136–138].

### NF-κB in lymphocyte-mediated renal inflammation

In addition to mediating inflammation of the innate immune system, NF-κB has a crucial role in regulating the autoimmune and inflammatory functions of T and B cells [139]. Canonical NF-κB pathway is required for the generation of Th17 cells from naïve CD4^+^ T cells [51, 52]. Although noncanonical NF-κB pathway is not important for Th17 differentiation, it is crucial for the pathological effector function of Th17 cells in mediating inflammation [55]. Of note, the Th17 subset of CD4^+^ inflammatory T cells has been implicated in the pathogenesis of renal inflammation [140, 141]. Experimental renal injury in animal models is associated with infiltration of effector T cells, including Th17 cells [142]. Kidney infiltration with Th17 cells has also been found in human patients with kidney diseases [141]. In a mouse model of antigen-specific glomerulonephritis, in which the antigen ovalbumin is planted on the glomerular basement membrane of *Rag1*-knockout mice, injection of ovalbumin-specific Th1 or Th17 cells induces proliferative glomerulonephritis [143]. Genetic evidence for the involvement of Th17 cells in renal inflammation was obtained using animal models deficient in the IL-17 signature cytokine IL-17 or the Th17-maintence cytokine IL-23 [144–146]. Given the crucial role of NF-κB pathways in the generation and effector function of Th17 cells, the pro-inflammatory functions of NF-κB in renal diseases likely involve inflammatory T cells.

B cells have a central role in the pathogenesis of lupus nephritis and IgA nephropathy [147, 148]. Both canonical and noncanonical NF-κB pathways are crucial for the survival and function of B cells [149]. The BAFF/BAFFR signaling system provides a strong stimulus for the activation of noncanonical NF-κB pathway and also stimulates additional survival pathways, including the canonical NF-κB and PI3 kinase pathways [13, 14, 149]. Thus, under physiological conditions, BAFF maintains the survival of B cells and is required for B cell maturation in the spleen [150]. However, deregulated production of BAFF has been linked to the pathogenesis of lupus nephritis and IgA nephropathy [151, 152]. Patients with lupus nephritis and IgA nephropathy have elevated level of serum BAFF, which is associated with clinical severity of the diseases [153–155]. Transgenic mice overexpressing BAFF have B cell hyperplasia and autoimmune manifestations, including nephritis and IgA nephropathy-like symptoms [110]. In line with this finding, mice deficient in a negative regulator of the noncanonical NF-κB pathway, TBK1, also have aberrant production of IgA and develop nephropathy-like symptoms [39]. A monoclonal antibody targeting soluble BAFF, belimumab, has been approved for the treatment of lupus with promising potential for the treatment of autoimmune kidney diseases like lupus nephritis [152].

## Concluding remarks

NF-κB has been well established as a pivotal mediator of inflammation, although its role in mediating inflammation in specific organs is less well understood. Nevertheless, accumulating studies suggest the involvement of NF-κB in the pathogenesis of renal inflammatory diseases. NF-κB is activated in both human patients with kidney diseases and animal models of renal inflammation and injury. NF-κB appears to mediate renal inflammation in different cell types, including renal cells, innate immune cells, and lymphocytes. It is thus clear that targeting NF-κB signaling pathway represents an attractive therapeutic approach in renal disease treatment. However, global inhibition of NF-κB may cause severe side effect, since NF-κB is required for normal immune responses and cell survival. Understanding the mechanism that underlies pathological activation of NF-κB in renal diseases is crucial for designing more specific and effective therapeutic agents.

## Abbreviations

NF-κB: nuclear factor kappa B
IKK: IκB kinase
NEMO: NF-κB essential modulator
TAK1: TGFβ-activated kinase 1
MAP3K: MAP kinase
K63: lysine 63
HOIL-1: heme-oxidized IRP2 ubiquitin ligase-1
HOIP: HOIL-1-interacting protein
SHARPIN: SHANK-associated RH domain-interacting protein
TNFR: TNF receptor
NIK: NF-κB inducing kinase
LTbR: lymphotoxin beta receptor
BAFF: B cell-activating factor belonging to the TNF family
BAFFR: BAFF receptor
RANK: receptor activator of nuclear factor-kappaB
TWEAK: tumor necrosis factor-related weak inducer of apoptosis
PAMP: pathogen-associated molecular pattern
DAMP: damage-associated molecular pattern
PRR: pattern-recognition receptor
TLR: toll-like receptor
RLR: RIG-I like receptor
NLR: NOD-like receptor
CLR: C-type lectin like receptor
Th: T helper
Tfh: T follicular
Treg: T regulatory
IFNγ: interferon gamma
SLE: systemic lupus erythematosus
siRNA: small interfering RNA
PDTC: pyrrolidine dithio-carbamate ammonium
ABIN1: A20-binding inhibitor of NF-κB1
Fn14: fibroblast growth factor-inducible 14
AKI: acute kidney injury
